# PET/MRI enables simultaneous *in vivo* quantification of β-cell mass and function

**DOI:** 10.7150/thno.33410

**Published:** 2020-01-01

**Authors:** Filippo C. Michelotti, Gregory Bowden, Astrid Küppers, Lieke Joosten, Jonas Maczewsky, Volker Nischwitz, Gisela Drews, Andreas Maurer, Martin Gotthardt, Andreas M. Schmid, Bernd J. Pichler

**Affiliations:** 1 Werner Siemens Imaging Center, Department of Preclinical Imaging and Radiopharmacy, Eberhard Karls University Tübingen, Tübingen, Germany; 2 Forschungszentrum Jülich GmbH, Central Institute of Engineering, Electronics and Analytics, Jülich, Germany,; 3 Radboud University Medical Center, Department of Radiology and Nuclear Medicine, Nijmegen, The Netherlands; 4 Experimental Diabetology, Phamacology, Pharmaceutical Institute, Eberhard Karls University Tübingen, Tübingen, Germany; 5 Cluster of Excellence iFIT (EXC 2180) "Image-Guided and Functionally Instructed Tumor Therapies", University of Tübingen, Germany

**Keywords:** PET, MRI, PET/MRI, β-cell function, β-cell mass, VDCC, GLP-1R.

## Abstract

Non-invasive imaging of β-cells represents a desirable preclinical and clinical tool to monitor the change of β-cell mass and the loss of function during pre-diabetic stages. Although it is widely accepted that manganese (Mn) ions are actively gated by voltage-dependent calcium channels (VDCC) in response to glucose metabolism, little is known on its specificity *in vivo* for quantification of islet β-cell function using Mn and magnetic resonance imaging (MRI). On the other hand, glucagon-like-peptide-1 receptor (GLP-1R) represents a validated target for the estimation of β-cell mass using radiolabeled exendin-4 (Ex4) and positron emission tomography (PET). However, a multiparametric imaging workflow revealing β-cell mass and function quantitatively is still missing.

**Methods:** We developed a simultaneous PET/MRI protocol to comprehensively quantify *in vivo* changes in β-cell mass and function by targeting, respectively, GLP-1R and VDCC coupled with insulin secretion. Differences in the spatial distribution of Mn and radiolabeled Ex4 were monitored overtime in native and transgenic pancreata, characterized by spontaneous pancreatic neuroendocrine tumor development. Follow-up with mass spectrometry imaging (MSI) and autoradiography allowed the ex vivo validation of the specificity of Mn and PET tracer uptake and the detection of endogenous biometals, such as calcium and zinc, throughout the endocrine and exocrine pancreas.

**Results:** Our *in vivo* data based on a volumetric PET/MRI readout for native pancreata and insulinomas connects uptake of Mn measured at early imaging time points to high non-specific binding by the exocrine tissue, while specific retention was only found 24 h post injection. These results are supported by cross-validation of the spatial distribution of exogenous ^55^Mn and endogenous ^44^Ca and ^64^Zn as well with the specific internalization of the radiolabeled peptide targeting GLP-1R.

Conclusion: Simultaneous PET/MR imaging of the pancreas enabled the comprehensive *in vivo* quantification of β-cell function and mass using Mn and radiolabeled Ex4. Most important, our data revealed that only late time-point measurements reflect the Mn uptake in the islet β-cells, while early time points detect non-specific accumulation of Mn in the exocrine pancreas.

## Introduction

Diabetes is a metabolic disease with a rising incidence across the globe 1. Despite the heterogeneity of processes leading to the onset of type-2 diabetes (T2D), defects in β-cell function, involving the impairment of insulin production and the secretion, have a central role in the establishment of insulin resistance and glucose intolerance 2-4. Transient elevation of blood glucose levels results in the enhancement of β-cell activity leading to functional exhaustion and, ultimately, to the failure of β-cells. A long debated issue is addressing the interaction and priority of β-cell mass and function during the early and late stages of diabetes progression 5-7. Thus, comprehensive knowledge of the natural course of diabetes onset leading to loss of β-cell mass and function would improve the chances of early medical intervention.

In the last decade, an impressive effort was put into the development of noninvasive imaging technologies to quantify the loss of β-cell mass and function *in vivo* 8-11. Magnetic resonance imaging (MRI) and nuclear imaging techniques such as single-photon emission computed tomography (SPECT) and positron emission tomography (PET) offer valid platforms for the translation of a new diagnostic approach, due to their elevated tissue penetrance and detection sensitivity.

Despite the low abundance of endocrine islet cells, the high expression level of glucagon-like-peptide-1 receptor (GLP-1R) on the surface of β-cells enables their specific detection and quantification in the native pancreas as demonstrated by the use of radiolabeled Exendin-4 (Ex4) 12-16, a potent GLP-1R agonist 17. While PET imaging of GLP-1R reliably reveals β-cell mass *in vivo*, recent studies showed that manganese-enhanced MRI (ME-MRI) provide quantitative assessment of β-cell function 18-21. Mn uptake by the pancreatic islets is coupled to the activity of voltage-dependent calcium channel (VDCC) and insulin secretion. Although the specificity of Mn for VDCC was confirmed previously *in vitro* on isolated islets using glucose or VDCC blockers 20, 22, little is known on the actual contribution of exocrine and endocrine pancreas on the overall uptake of Mn *in vivo* 23.

Using combined PET/MR, we aimed to simultaneously measure β-cell mass and function in a single imaging session, by targeting GLP-1R and VDCC using [^64^Cu]Ex4 and MnCl_2_. Such a dual imaging approach enables the correlation of the comprehensive imaging information at high spatial and temporal resolution. A focus of our work was also on the assessment of the pharmacokinetics of both, [^64^Cu]Ex4 and Mn and thus, the determination of the optimal imaging time point for accurate quantification of β-cell mass and function *in vivo*. In particular we aimed to address the contribution of exocrine and endocrine pancreas to the overall uptake of Mn and the PET tracer.

Therefore, we have longitudinally imaged healthy and transgenic RIP1-Tag2 mice that, in a well-defined multistep process, develop neuro-endocrine pancreatic insulin-producing tumors (insulinomas) derived from proliferating β-cells 24, 25. For the first time, we cross-validated the spatial co-localization of Ex4 and Mn within the endocrine and exocrine pancreas by combining autoradiography of the PET tracer and imaging of biometals using mass spectrometry imaging (MSI). Thus, the identification of the endocrine pancreas through the endogenous levels of zinc, calcium and the specific accumulation of PET tracer enabled us to address the specificity of the elevated Mn uptake at early and late retention time points, 1 h and 24 h after the injection.

Our studies revealed that the initial uptake of Mn was not specific for β-cell function mostly due to the high background signal from the exocrine pancreas. However, we found that Mn is retained specifically within the endocrine pancreas at later time points as demonstrated *in vivo* by the positive correlation between PET and MRI signal and by the *ex vivo* co-localization of biometals, Mn and the PET tracer. Finally, we proposed that only late time point imaging of Mn is a valid biomarker for β-cell function.

## Results

### Elevated exocrine uptake of Mn at early time point limits *in vivo* detection of pancreatic islets and insulinomas

**Figure 1:**
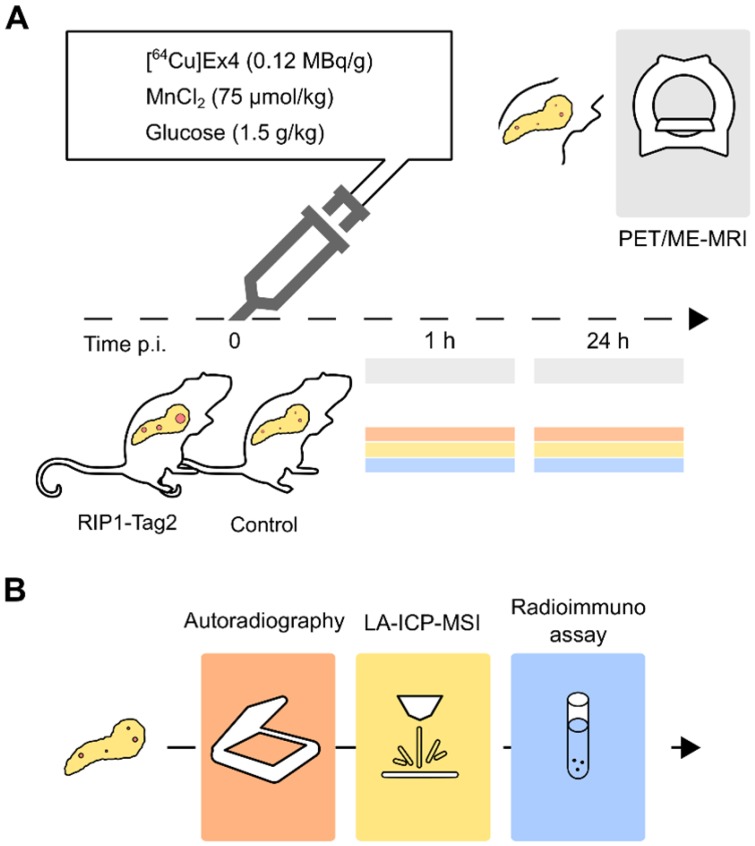
** Experimental imaging workflow *in vivo* followed by *ex vivo* analysis to cross-validate the pancreatic distribution of [^64^Cu]Ex4 and Mn. (A)** *In vivo* PET/ME-MRI aimed to simultaneously quantify β-cell mass and function: we acquired simultaneously or consecutively PET/MR images after 1 h and 24 h the co-injection of [^64^Cu]Ex4 and MnCl_2_. **(B)** In order to confirm our *in vivo* results, we cross-validated the specificity of the PET tracer and Mn in pancreas sections using autoradiography and MSI of divalent metals. On a separate group of mice we assessed the glucose-dependent insulin secretion at early (1 h) and late time points (24 h) by radioimmunoassay of insulin from isolated islets.

Groups of control and transgenic 13-weeks (wk)-old RIP1-Tag2 mice (n=3/3) with established insulinomas were measured between 0- 1 h post injection using a simultaneous PET/MR setup (Table 1). As illustrated in Fig. 1, *in vivo* PET/ME-MRI was performed after the intravenous (i.v.) co-injection of imaging probes, [^64^Cu]Cu-NODAGA-^40^Lys-Exendin-4 ([^64^Cu]Ex4) and MnCl_2_:Bicine solutions, followed by *ex vivo* analysis of pancreas sections.

**Figure 2:**
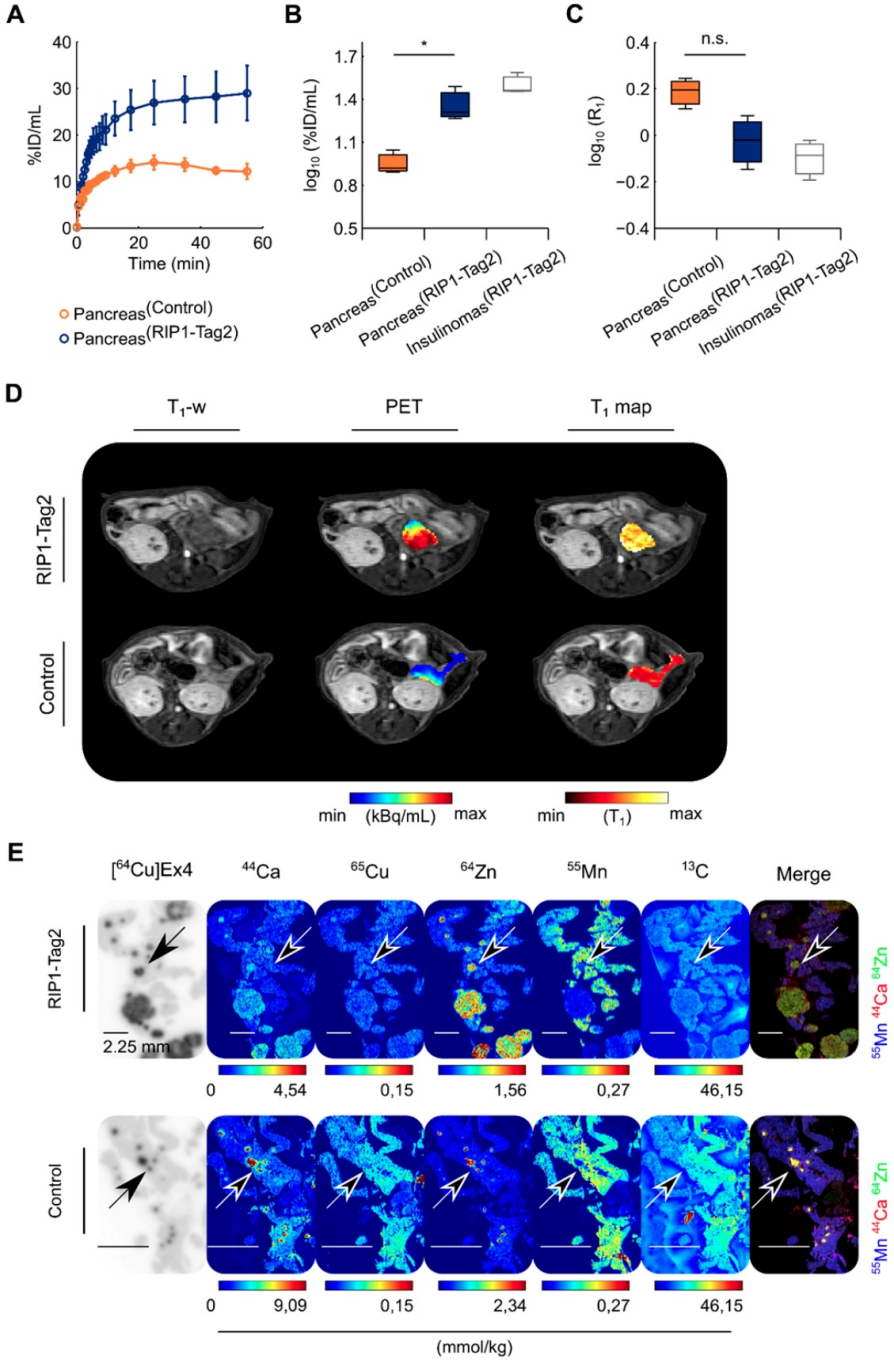
** The elevated uptake of Mn measured at the early time point (1 h) in the pancreas is not specific for the endocrine pancreas.** A group of 13-wk-old RIP1-Tag2 and control mice (n=3/3) were dynamically measured using a simultaneous PET/MRI setup followed by autoradiography and MSI after 1 h of the co-injection of the PET tracer and MR-contrast agent. **(A)** Dynamic tracer uptake PET curves display the %ID/mL of [^64^Cu]Ex4 plotted for control pancreata (dark orange circles) and transgenic RIP1-Tag2 pancreata (dark blue circles). The respective values for the detected pancreatic insulinomas (Insulinomas^RIP1-Tag2^) are shown as reference (light grey boxes). Differences between the two groups (Pancreas^Control^; Pancreas^RIP1-Tag2^) were calculated using two sample Student's *t*-test for the **(B)** PET (*t*(3.67)=4.32, *p=0.01) and **(C)** ME-MRI quantification (*t*(3.18)=-2.75, *p*=0.06). The boxes represent the 1^st^, 2^nd^ and 3^rd^ quartile; the length of the whiskers is equal to 1.5 times the interquartile range above and below the 1^st^ and the 3^rd^ quartile, respectively. **(D)** A representative co-registered PET/MR image shows the relationship between the uptake of the PET tracer and Mn in the region of pancreata of control and RIP1-Tag2 mice. **(E)** The figure illustrates the autoradiography of [^64^Cu]Ex4 and MSI of ^44^Ca, ^65^Cu, ^64^Zn, ^55^Mn and ^13^C obtained from the same pancreas sections in both the groups. Scale bars are settled at 2.25 mm. Merged images display the normalized levels of ^55^Mn (blue channel), ^44^Ca (red channel) and ^64^Zn (green channel). Diffuse spot-like areas identified by elemental imaging (yellow spots) and autoradiography (dark spots) indicated with the black arrows. Merged elemental images indicate the high concentration of exogenous ^55^Mn (blue channel) levels in the exocrine pancreas compared to the islet clusters and insulinomas positive for the accumulation of PET tracer.

Dynamic PET curves indicated a stable pancreatic uptake after 20 min until 1 h after the tracer injection in both groups (Fig. 2A). The regions of insulinomas detected in the same group of RIP1-Tag2 mice are reported only as reference. The statistical analysis revealed a tracer uptake significantly higher (*t*(3.67)=4.32, *p=0.01) in the pancreata of 13-wk-old RIP1-Tag2 mice compared to the control group (Fig. 2B), with a factor of 1.4 and 1.6 times higher for the pancreata and the detected insulinomas respectively. We validated our statistical analysis by testing the distribution of all the PET and MRI data (Fig. S1A-B, Table 1) we collected in our studies. In comparison with the PET measurements, analysis of quantitative maps of the longitudinal relaxation time (T_1_), reflecting Mn uptake, revealed the highest accumulation of contrast agent in the pancreas of control animals compared to the RIP1-Tag2 mice (*t*(3.18)=2.75, *p*=0.06) and insulinomas, respectively (Fig. 2C). As shown in Fig. 2D, we co-registered the PET images with the volumetric T_1_-weighted (T_1_-w) images and the T_1_ maps in order to define the anatomical boundaries of the pancreas and insulinomas. The analysis of the relation between the effect of Mn (R_1_=1/T_1_) and the uptake of [^64^Cu]Ex4 (%ID/mL) produced negative correlation coefficients in both the groups (Fig. S2A). Similarly, the voxel-wise correlation analysis of pancreata revealed a moderate negative linear relationship between the PET tracer and Mn uptake (Fig. S2B) in the pancreata of RIP1-Tag2 mice in contrast to the correlation coefficients obtained from the control group.

As supporting evidences for our *in vivo* results, we performed autoradiography followed by MSI of the pancreas sections (Fig. 2E). The coefficients of determination (R^2^) calculated for each separated animal indicated that the PET tracer uptake is a positive predictor of the islet diameter in both groups (Fig. S3A-C). In contrast to the distribution of ^65^Cu and ^13^C, the distribution of endogenous ^44^Ca and ^64^Zn showed excellent localization with the PET tracer uptake (Fig. 2E). The analysis of exogenous ^55^Mn was performed to cross-validate the specific localization of Mn-based contrast agent with the autoradiography of the PET tracer. However, the merged elemental images produced by overlaying of ^55^Mn, ^44^Ca and ^64^Zn levels revealed a higher concentration of ^55^Mn in the exocrine pancreas. In a separated study, we confirmed that the accumulation of radiotracer was specific for the pancreatic insulinomas found with elevated insulin content (Method S2-S3 and Fig. S4). Altogether, our results indicate that *in vivo* Mn imaging at early time points is not directly related to β-cell mass and function, since the elevated uptake of Mn in the pancreas is not specific for the receptor density of GLP-1R on the surface of islets β-cells neither to the high content of zinc and calcium, which are important for the storage and the secretion of insulin in the endocrine pancreas.

### Late time point Mn imaging revealed a specific intracellular transport in pancreatic islets and insulinomas

**Figure 3:**
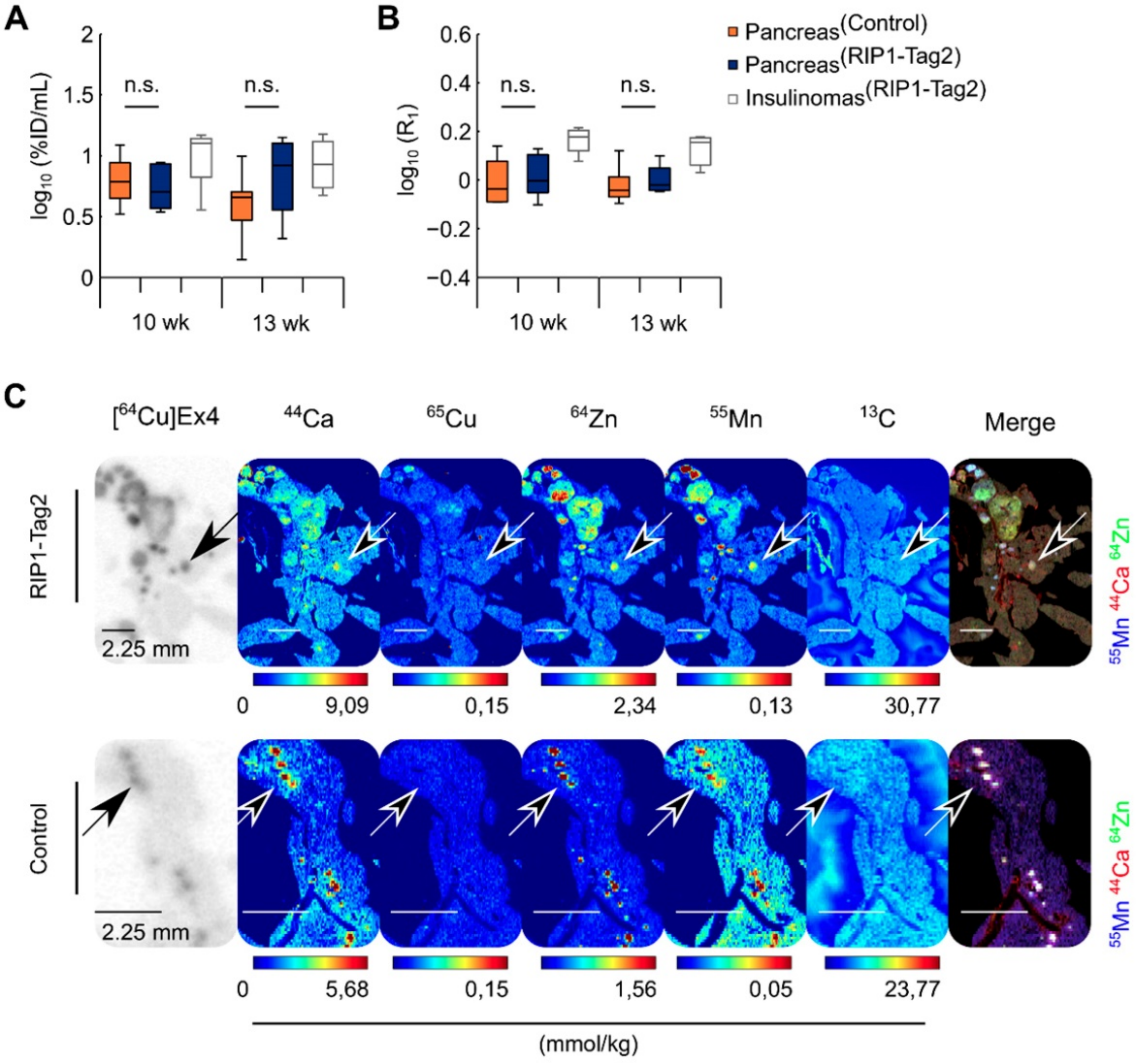
** The retention of Mn in the pancreas measured at late time points (24 h) is specific for the pancreatic islets and insulinomas.** RIP1-Tag2 and control mice (n=3/4) were measured at 10 and 13 wk of age using a consecutive PET/MRI setup. Autoradiography and MSI were performed after 24 h of the co-injection of the PET tracer and MR-contrast agent at the last imaging point. The boxes represent the 1^st^, 2^nd^ and 3^rd^ quartile of the pancreas of control and (Pancreas^Control^) RIP1-Tag2 mice (Pancreas^RIP1-Tag2^) as well as the reference values calculated from the detected pancreatic insulinomas in the same group of mice (Insulinomas^RIP1-Tag2^), longitudinally measured at 10 and 13 wk of age with quantitative **(A)** PET and **(B)** MR imaging; the length of the whiskers is equal to 1.5 times the interquartile range above and below the 1^st^ and the 3^rd^ quartile, respectively. **(C)** Autoradiography of [^64^Cu]Ex4 followed by MSI of ^44^Ca, ^65^Cu, ^64^Zn, ^55^Mn and ^13^C on the same tissue slice. The black arrows indicate the accumulation of PET tracer (dark spots) in the same tissue areas with high levels of ^55^Mn, ^44^Ca and ^64^Zn (white spots) shown in the merged elemental images and reflecting the spatial distribution of pancreatic islets and insulinomas of control and the transgenic RIP1-Tag2 mice.

Another group of control and RIP1-Tag2 mice (n=4/4) was monitored longitudinally during the progression of insulinomas (Table 1), with a consecutive PET/ME-MRI protocol and measurements at late time points (24 h) after PET tracer and MnCl_2_ injections. In contrast to the early imaging time point, only slight differences in the uptake of Mn and radiolabeled Ex4 were calculated between the groups of control and RIP1-Tag2 mice measured at 10 and 13 wk of age (Fig. 3A-B). This tendency was also reflected in the voxel correlation analysis in the pancreata of both groups, since we obtained only weak and not significant correlation coefficients (Fig. S2C-D). After the last imaging time point, we followed *ex vivo* the specific retention of the PET tracer and the content of exogenous ^55^Mn by the analysis of the pancreas sections of control and RIP1-Tag2 mice. MSI analysis showed co-localization of ^44^Ca and ^64^Zn with the autoradiography of the PET tracer for the late time point (24 h). In agreement with the *in vivo* data and in sharp contrast to the analysis at 1 h after Mn injection (Fig. 3C), the localization of high levels of ^55^Mn strikingly correlated with the endogenous levels of ^44^Ca and ^64^Zn as well as with the specific retention of the PET tracer. In contrast, only low levels of ^55^Mn were found in the exocrine pancreas. Thus, our results suggest that imaging of Mn at the late points correlate with β-cell mass and function due to the progressive wash-out of contrast agent by the exocrine pancreas and the specific retention of Mn by the endocrine β-cells and insulinomas. The regression analysis of autoradiography indicated a positive relationship between the late retention of the PET tracer and the islet size in both groups (Fig. S5A-C).

**Figure 4:**
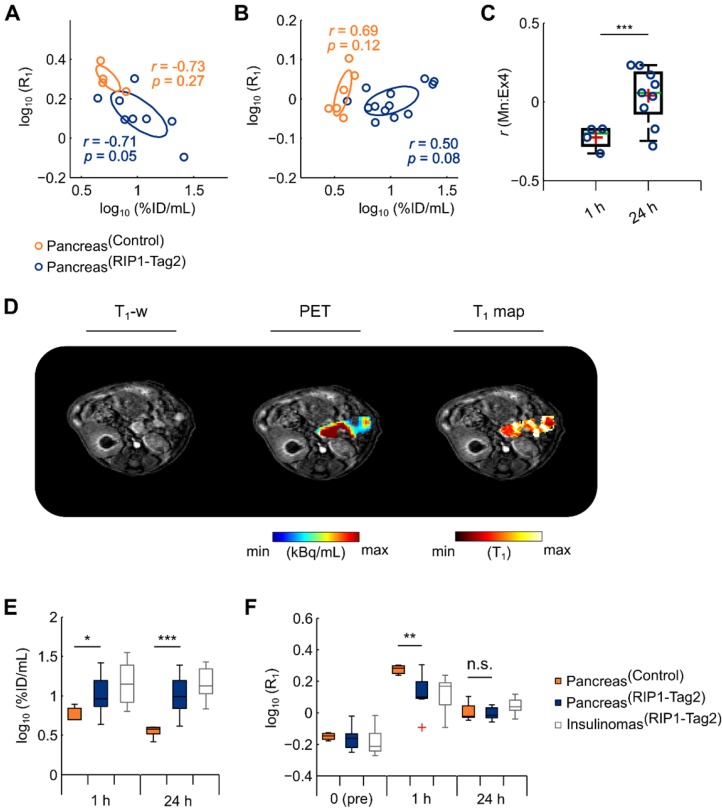
** High retention of Mn at late time points (24 h) correlates positively with the specific accumulation of PET tracer.** Correlation analysis of PET/MR was calculated by assessing the medians of log_10_(%ID/mL) values against the medians of log_10_(R_1_) of the pancreas measured at early **(A)** and late **(B)** time points in control (1 h: *r*=-0.73, *p*=0.27; 24 h: *r*=0.69, *p*=0.12; dark orange circles) and RIP1-Tag2 mice (1 h: *r*=-0.71, *p*=0.05; 24 h: *r*=0.50, *p*=0.08), respectively. **(C)** Differences between the *r* coefficients produced by each individual RIP1-Tag2 mouse (Pancreas^RIP1-Tag2^) measured either at early or late time points was calculated using a two sample Student's *t*-test (*t*(11.0)=4.55, ***p<0.001). Boxes represent the 1^st^, 2^nd^ (green line) and 3^rd^ quartile as well as the mean (red crosses) of each distribution. **(D)** The co-registered PET/MR images depict the quantification of PET tracer, T_1_ maps and the enhanced T_1_-w images from the abdominal region of a representative RIP1-Tag2 pancreas, measured at 24 h. The boxplot show the interquartile range distribution of the **(E)** medians log_10_(%ID/mL) and **(F)** log_10_(R_1_) before, at 1 h and 24 h after the injection of MR contrast agent; the outliers (red crosses) consist of data points with values higher than 1.5 times the interquartile range above and below the 1^st^ and the 3^rd^ quartile, respectively. Differences between the pancreas of control (Pancreas^Control^) and RIP1-Tag2 mice (Pancreas^RIP1-Tag2^) were calculated using two sample Student's *t*-test for quantitative PET and MR imaging.

In order to reproduce our *in vivo* results, we performed PET/ME-MRI with a consecutive setup and using an additional flip angle to increase the precision of T_1_ maps. Repeated measurements were acquired at early (1 h) (n=3/7) and late time point (24 h) (n=6/13) using a new group of control and RIP1-Tag2 mice. In the analysis, we pooled the data from the mice measured between 10 and 15 wk of age (Table 1). In agreement with our previous results, we found a moderate negative correlation between the elevated uptake of Mn at 1 h and the PET tracer in the pancreata of RIP1-Tag2 (*r*=-0.71, *p*=0.05) and control mice (*r*=-0.73, *p*=0.27) (Fig. 4A). We also observed numerous weak to moderate negative coefficients from the voxel-wise correlation analysis throughout the pancreata of RIP1-Tag2 mice (Fig. 4C). This tendency was clearly reversed at late time points and resulted in a positive correlation between PET tracer and Mn uptake within the groups of transgenic (*r*=0.50, *p*=0.08) and control mice (*r*=0.69, *p*=0.12) (Fig. 4B). This outcome was also reflected by the analysis of voxels as revealing moderate and positive coefficients (Fig. 4C) from the pancreata of RIP1-Tag2 mice. A representative image reflecting the levels of Mn and the PET tracer uptake is illustrated in Fig. 4D.

Differences in the uptake of radiolabeled Ex4 indicate %ID/mL was significantly higher in the pancreata of RIP1-Tag2 mice at early (*t*(9.94)=2.66, *p=0.02) and late time points (*t*(16.26)=5.89, ***p<0.001) (Fig. 4E, Table 2). In agreement with the previous studies, the uptake of Mn measured at early time points was significantly lower in the transgenic pancreata (*t*(9.63)=-3.44, **p=0.006) and the detected insulinomas compared to the healthy tissues, while we found only slight differences between the groups at later time points (*t*(6.89)=-0.78, *p*=0.47). Interestingly, the concentrations of contrast agent ([Mn]) estimated *in vivo* were approximatively 2 times lower in the detected insulinomas than in the pancreas of control mice (Table S1) at early time points, which agree well with the measurements of ^55^Mn *ex vivo*. Those results confirmed our previous *ex vivo* analysis, as we observed a strong retention of Mn from the pancreatic insulinomas and the native islets overtime.

We investigated the role of glucose on the elevated uptake and late retention of Mn in the pancreata at the early and late measurement time points on another group of mice (Table 1). Thus, on a separate group of control and RIP1-Tag2 mice we performed MSI and autoradiography without previously challenging the mice with glucose. Similarly to our previous experiments, we found elevated uptake of exogenous ^55^Mn in the exocrine pancreas compared to the endocrine pancreas, while we found high levels of endogenous ^44^Ca and ^64^Zn which were also positive for the specific accumulation of [^64^Cu]Ex4 (Fig. S6). The follow-up of the late accumulation of Mn revealed a specific retention of ^55^Mn in the native islets and insulinomas as observed from merged elemental images of ^55^Mn, ^64^Zn and ^44^Ca and the accumulation of PET tracer targeting GLP-1R.

### The physiological response of islets is not impaired by early and late retention of Mn in the pancreas

By monitoring the glycemic status of the previous group of mice (Table 1), we found a significant decrease in the blood glucose levels from the group of transgenic RIP1-Tag2 mice (Fig. S7A-B).

The chronic exposure to high concentrations of Mn can potentially lead to cytotoxic effects on heart, liver and brain 26. We hypothesized that the early and late retention of Mn might impair the physiological integrity of the glucose-dependent insulin response of islet cells. To investigate the physiological function of the islets, glucose-dependent insulin secretion was measured *in vitro* 1 or 24 h after a single systemic *in vivo* administration of MnCl_2_:Bicine solution (75 µmol/kg) on a new group of mice (Table. 1, Group 4). Secretion assays were performed on isolated islets, followed by quantification of insulin content using radioimmunoassay. The response to glucose of each individual mouse was assessed by calculating the enhancement of insulin secretion between basal (3 mM) and stimulating glucose concentration (15 mM).

By evaluating the enhancement of insulin secretion from the islets of 8-wk old control and RIP1-Tag2 mice, we found levels of insulin 10-20 times higher in response to stimulating glucose conditions in both groups (Fig. S8A-B). At this tumor stage, we did not observe clear differences in the distribution of islet size between the two groups (Fig. S8C). Similar physiological indexes of insulin response were found at early (1 h) and late time point (24 h) in both groups, suggesting that the insulin secretion response dependent to glucose was not affected by the accumulation of the contrast agent in the pancreas. A further experiment was performed to evaluate the secretion of insulin in response to glucose from the islets of 13-wk-old control and transgenic mice, which are characterized by late tumor development (Table 1). Compared with the enhancement in the insulin secretion induced by glucose from the islets of mice at early tumorigenic stages, we found high insulin secretion levels at both basal and stimulating glucose conditions (3-15 mM), which might be due to impairment in the physiological response to glucose (Fig. S9A-C).

## Discussion

In the last decades, a considerable effort has been taken developing imaging methods to assess specifically the β-cells in the native and diseased pancreas. The GLP-1R has been shown to be a promising target for the quantification of β-cell mass using nuclear imaging techniques and was investigated as highly promising candidate to monitor the progressive loss of β-cell mass in diabetes 16. However, recent evidences suggested that low β-cell mass is not necessarily accompanied by diabetes 4, 5. Indeed, defects in β-cell function, such as synthesis, storage and glucose-dependent secretion of insulin, seem to play a pivotal role for the establishment of pre-diabetic stages. The clinical tests assessing the plasma levels of glucose, insulin and C-peptide levels can measure indirectly the secretory capacity of β-cells, although they cannot discriminate the changes in the synthetic workload placed on each β-cell during the progressive loss of functional β-cell mass. To partially bridge this gap, Mn imaging is a promising tool, since the uptake of Mn relies on the activity of VDCC and, thus, with the secretory capacity of islet β-cells. Previous imaging studies validated *in vivo* the specificity of Mn for the pancreatic β-cells using VDCC blockers or streptozotocin (STZ)-treated mice 18-20. However, controversial results showed that changes in the vascularity of the pancreas, rather than β-cell destruction, might lead to a decrease in perfusion and wash-out of Mn in STZ-mice 19. Thus, the specificity of the overall Mn for the pancreatic islets as well as the contribution of the exocrine pancreas on the overall uptake of Mn *in vivo* remained unclear 23.

Our main focus was to determine the feasibility of a PET/MRI approach to quantitatively correlate *in vivo* β-cell mass and function by targeting simultaneously the receptor density of GLP-1R on the surface of β-cells and the activity of VDCC, being coupled with the glucose-dependent insulin secretion. Our imaging methods were developed and validated measuring the whole pancreas of healthy and transgenic mice developing cell and tissue specific insulin-secreting tumors. We showed that the uptake of radiolabeled Ex4 was higher in the entire pancreas of RIP1-Tag2 mice developing overtime pancreatic insulinomas by hyperplasia of β-cells expressing GLP-1R. *Ex vivo* analysis also confirmed the specificity of the PET tracer for β-cell mass since we showed that the signal from autoradiography was linearly related with the islet cluster dimensions and with the spatial localization of the endogenous levels of calcium and zinc, representing β-cells in the pancreas. The high content of zinc and calcium can be linked to the dense zinc-insulin complexes that are stored in the granules of β-cells. The influx of calcium in the cytosol of β-cells mediates the secretion of insulin granules and is promoted by the metabolism of glucose 27. Due to the limitation of mass spectrometry imaging and autoradiography in resolving the localization of probes at the cellular level, we cannot exclude that both Mn and radioactive Ex4 could potentially accumulate in other islet cell types expressing VDCC and GLP-1R. Noteworthy, the islet cytoarchitecture in rodents consists in large majority of β-cells (85-90%) arranged in a dense 'core' surrounded by a 'mantle' populated by α-cells (10-15%) and minor percentages of the other cell types. In a recent study, Brom et al. showed that the uptake of radiolabeled exendin-derivatives is not influenced by the presence of α-cells expressing GLP-1R 15.

Previous work demonstrated the mechanism and the specificity of tracers targeting GLP-1R for insulin-positive β-cells in the pancreas 13, 28, 29. Several studies showed that the specific accumulation of radiolabeled exendin-derivatives enabled the detection of islet β-cells clusters in control and diabetic murine models even in viable cells with insulin content below the detection limits of current immunohistochemical methods 13, 30, 31. Here, we demonstrated that the uptake of our PET tracer by the pancreatic insulinomas is colocalized with insulin-secreting cells expressing GLP-1R.

In the same cohort of healthy and transgenic RIP1-Tag2 mice, we elucidated the specificity of Mn used as MR contrast agent to probe β-cell function in the different compartments of the pancreas between early and late uptake. At the early time point, 1 h post injection, *in vivo* quantification of T_1_ maps, reflecting the uptake of Mn, was inversely related to the PET data, within the groups and also within each pancreatic region; the uptake of Mn in the detected insulinomas was lower compared to the healthy tissue, which is in contrast with the elevated secretory capacity measured from the isolated islet β-cells of RIP1-Tag2 mice.

Our *ex vivo* analysis confirmed that the elevated Mn uptake in the pancreas at the early time points is not related to β-cell mass and function as observed from the negative spatial correlation of exogenous ^55^Mn with the uptake of the radiolabeled Ex4, reflecting β-cell mass, and the high endogenous levels of calcium and zinc. In accordance with other studies on the essential elements in the pancreas, the quantification of endogenous levels of zinc and calcium are highly sensitive in detecting low density islet cells diffused throughout the pancreas sections 32. Recently, a zinc-responsive MR-contrast agent was shown to quantify *in vivo* the secretory capacity of islet cells releasing insulin-zinc complexes in the extracellular space in response to glucose 33.

We demonstrated that, at an early time point, Mn uptake was not specific for the pancreatic islets, however, importantly our results revealed that at 24 h post injection Mn accumulated specifically in the islet cells of native pancreas and insulinomas. In fact, retained accumulation of Mn at the later time point was in good agreement with the signal from PET. The positive relationship between PET and MRI signals suggests that an increase in the β-cell mass in insulinomas could also be related to a respective increase of β-cell function and, thus, to the capacity of the pancreas to secrete insulin. More importantly, we confirmed our *in vivo* results by addressing the specific co-localization of the late retention of Mn with the PET tracer and the divalent metals important for the metabolism of insulin. Furthermore, we reproduced our results using a dedicated PET/MRI protocol as we monitored the changes overtime in the specificity of PET/MRI signal between early and late time points. The late retention of Mn by the endocrine insulinomas correlated with the quantification of PET tracer throughout the whole pancreata and it was in good agreement with the glucose levels in the blood of RIP1-Tag2 mice.

Due to the potential toxicity of Mn-compounds, delivering low Mn concentration in the tissue of interest represents a major challenge to safely translate Mn imaging methods into the clinic 26. In this regard, our observations suggested that the accumulation of Mn did not impair the integrity of the glucose-dependent insulin response in islets isolated between the early and late time points. Here, we did not investigate the effect of glucose and anesthesia on the initial perfusion of contrast agent; however, the elemental images combined with autoradiography revealed that the initial stimulation with glucose did not record drastic changes in the overall distribution of the contrast agent or the PET tracer at early or late time points.

Retrospective clinical studies showed delayed MRI at 24 hours post contrast agent injection, using [Mn^2+^-*N*,*N'*-dipyridoxylethylenediamine-*N*,*N'*-diacetate-5,5*'*-bis-(phosphate)] (MnDPDP) in patients with endocrine tumor metastasis, resulted in a lower contrast enhancement in the liver parenchyma while the uptake in the lesions remained the same compared to an early imaging time point 34, 35. This is in line with our findings, however, we concentrated on the endocrine and exocrine pancreas and specific β-cell imaging. Interestingly Botsikas et al. showed a lower uptake of Mn in the pancreas of diabetes patients, compared to healthy controls, for images acquired immediately after the injection of MnDPDP (1-4 h) 36. Since our data show that Mn is not specific at an early time point, the lower Mn signal enhancement in the pancreas of diabetic patients might be related to the vascularity of pancreas, rather than β-cell mass and function.

In conclusion, our studies revealed that only late retention of Mn can be associated with the secretory function of pancreas, due to the high background signal from the exocrine pancreas observed during the early time points. Using a simultaneous PET/MRI method we demonstrated the positive correlation between radiolabeled Ex4, reflecting β-cell mass, and the specific retention of Mn throughout the endocrine and exocrine pancreas *in vivo*. In addition, we validated *ex vivo* the co-localization of essential biometals, Mn and the PET tracer in native and diseased pancreatic β-cells. Our observations on Mn uptake, in close relationship with previous clinical reports on MnDPDP, indicate that our measurements on β-cell function can be translated into the clinic. Finally, PET/MRI has the potential to quantitatively estimate critical indexes regarding the relationship between β-cell mass and function in diabetes patients.

## Methods

### Radiolabeling of [^64^Cu]NODAGA-^40^Lys-Exendin-4

[^64^Cu]CuCl_2_ (100 MBq in 0.1 M HCl) was neutralized with 1.5X its volume of ammonium acetate buffer (pH 6). To this solution was added NODAGA-^40^Lys-Exendin-4, 4 µg in 4 µL of deionized water. After mixing, the solution was incubated at 42 °C for 20 minutes (min). RadioTLC (Polygram Sil G/UV_254_) run using a citrate buffer (pH 5) as the mobile phase was used to determine the labeling efficiency of the reaction (typically 90-95% with < 2% free ^64^Cu). The reaction solution was formulated for injection with PBS buffer (0.1% Tween 20) to a final volume of 400 µL.

### RIP1-Tag2 mouse model

Transgenic RIP1-Tag2 mice carrying the Simian Virus 40 large T antigen (Tag2) controlled by the Rat Insulin promotor (RIP1) develop β-cell derived insulinomas at high reproducibility starting from hyperplasia at early stages (5 wk of age) until the formation of solid tumors (14 wk of age)^24^, ^25^ with symptomatic blood glucose levels. Progression of hypoglycemia was monitored using a blood analyzer device (HemoCue Hb 201+, HCE, United Kingdom) during the progression of insulinomas. Animals were sacrificed when severe hypoglycemia occurred (< 30 mg/dL). Cohorts of in-house bred transgenic RIP1-Tag2 and age-matched littermate control mice with a C3H/FeJ background (Table 1) were measured longitudinally over the progression of insulinomas (10 to 13 wk of age). The animals were kept under sterile environment conditions in isolated ventilated cages at approximately 22 °C room temperature, 54% of relative humidity and 12 h light/dark cycle: food and water were provided *ab libitum*.

All animal experiments were carried out in accordance to the German Animal Welfare Law and accordingly with the permission approved by the responsible local authorities (Regierungspräsidium Tübingen; DE).

### PET/ME-MRI protocol

In brief, mice were fasted for 4-6 hours and anesthetized with 1-2% isoflurane/O_2_ gas mixture (Vetland, Louisville, KY, USA) before the *in vivo* measurements. A tail vein catheter was initially placed for the co-injection of 50 µL of tracer solution (0.12 MBq/g bodyweight) and 50 µL of MnCl_2_:Bicine solution (75 µmol/kg body weight). The animals were placed in supine position on the bed in order to attenuate the motion artifacts rising from the respiratory movements. Thus, [^64^Cu]Ex4 and MnCl_2_ solution were co-injected i.v. as a bolus a few seconds after the beginning of dynamic PET measurements. An additional i.v. injection of glucose solution (1.5 g/kg) was performed five minutes after the PET tracer and MR contrast agent injection to enhance the metabolic uptake of Mn by the pancreas. PET quantification was performed by 20-min static frames acquired simultaneously with 7T MRI scanner using a compatible PET-insert or consecutively using an Inveon-PET scanner (Inveon Dedicated PET, Siemens Healthineers, Knoxville, TN, USA). MR images were acquired using a mouse body quadrature volume RF-resonator with an inner diameter of 40 mm (MT0205, Bruker) on a 7 T Preclinical MR scanner (BioSpec 70/30, Bruker BioSpin MRI GmbH, Ettlingen, DE). During the acquisition, we monitored the breathing with an MR-compatible breathing sensor. The body temperature was maintained at 37 °C using a water-based warming pad.

### MR pulse sequences

*In vivo* MR measurement started with the acquisition of a rapid acquisition with relaxation enhancement (RARE) turbo-spin-echo (TSE) sequence with a large field of view to perform whole-body imaging with the following MR parameters: fat suppression module, repetition time (TR)/echo time (TE)=800/30.8 ms, respectively, field of view (FoV)=64×32×23 mm^3^, matrix size 256×128, one average, bandwidth (BW)=75 kHz, RARE Factor (RF)=16 and an isotropic resolution of 0.25 mm. Then, we acquired consecutive RF-spoiled and slab selective gradient-echo (GRE) pulse sequences with short TR and keeping at the minimum the TE and using the following MR parameters: TR/TE=10/1.9 ms, FoV=34×34×17 mm^3^, matrix size 128×128, two averages, BW=75 kHz, Sharpness=7 and approximatively 3 min of acquisition time per scan. Quantification of T_1_ was calculated by the acquisition of either two flip angles (4°, 22°), 0.27×0.27 mm^2^ of in-plane resolution and 0.53 mm of slice thickness, or three flip angles (4°, 14°, 27°) with an isotropic resolution of 0.27 mm as described in Table 1.

### PET image reconstruction

PET images were reconstructed with a 3D ordered subsets expectation maximization with maximum a posteriori algorithm (OSEM3D-MAP) implemented in Inveon Acquisition Workplace (Siemens Healthineers) with an image matrix size of 256×256×159, iteration 2, MAP iteration 18 and voxel size 0.38×0.38×0.8 mm.

### PET/ME-MRI quantification and correlation analysis

The volume of interest (VOI) for the entire pancreas (Pancreas^Control^; Pancreas^RIP1-Tag2^) and the detected insulinomas (Insulinomas^RIP1-Tag2^) were manually drawn in Inveon Research Workplace (Siemens Heathineers) after co-registration of the PET images. The T_1_-w images and the T_1_ maps were as well co-registered and used as anatomical references. The co-registered VOIs were exported and analyzed in MATLAB (R2013b; The MathWorks, Natick, MA, USA) using an in-house script. The percent injected dose per mL (%ID/mL) of the PET tracer was calculated voxel-wise by adjusting the radioactivity units (kBq/mL) for the injected activity and the half-life of ^64^Cu (*t*_1/2_=12.7 h). On the other hand, the longitudinal relaxation rate (R_1_=1/T_1_) was calculated from the respective region drawn on the T_1_ maps. For the quantification, we considered voxels with R_1_ values between 0.4 and 10 s^-1^. The estimated concentrations of Mn ([Mn]) were also calculated voxel-wise by the subtraction image acquired before and after the injection of MR-contrast agent and using the relaxivity (*r_1_*) of MnCl_2_:Bicine ([Mn]∝∆R_1_=(R_1*post*_-R_1*pre*_)/*r_1_*), which was calculated separately in a phantom study (Method S1 and Fig. S10). The indexes of [Mn] after 24 h were calculated using the overall mean of R_1_ obtained before and after 24 h, due to the different anatomical localization of the pancreata. Correlation and quantification of PET/MRI datasets was performed using means of the log_10_(%ID/mL) and log_10_(R_1_) of control and transgenic pancreas or voxel-wise using the individual region. The number of voxels of each region was downsampled to reduce the mismatched occurring after image co-registration. The new voxels were generated by averaging of a number of data points between 2 and 8 in the three dimensions.

### Autoradiography

The whole pancreata from control and transgenic RIP1-Tag2 mice were isolated after cervical dislocation under anesthesia. Then, the whole pancreata were embedded in O.C.T. (optimum cutting temperature) embedding compound (Sakura, Zoeterwonde, NL) and snap frozen at -20 °C. Subsequently, serial 20 µm cryosections were obtained using a Cryostat (Leica 1850, Leica GmbH, Wetzlar, DE) and exposed to a 35×43 cm Storage Phosphor Screen (445SI, Molecular Dynamics, Sunnyvale, CA, USA) at room temperature for 24 h and 48 h for animals sacrificed at 1 h and 24 h, respectively. Scanning of the phosphor imaging plate was performed using a Storm 840 scanner (STORM 840, Amersham Biosciences, Amersham, UK) with a spatial resolution of 50x50 µm^2^ and analyzed using ImageJ software 37 (US NIH, Bethesda, Maryland, USA). Thus, we calculated the main effect of the PET tracer uptake, expressed as islet-to-exocrine ratio to estimate the islet diameter identified as single spots throughout the pancreas sections in both groups (Fig. S3).

### LA-ICP-MS imaging

The autoradiography slides were scanned line-by-line at 60 µm laser spot size using an NWR 213 laser ablation system (New Wave Research, Fremont, CA, USA). The aerosol of the ablated tissue was transported via an argon gas flow through a transfer line to an Agilent 7900 ICP-MS (Agilent Technologies, Japan). The isotopes ^44^Ca, ^65^Cu, ^64^Zn,^ 55^Mn, ^13^C were monitored among the exocrine and the endocrine tissue in the healthy and insulinoma mice. Semi-quantitative evaluation was achieved by a calibration using spiked rat brain tissue standards. Image reconstruction was performed via an in-house software package IMAGENA 38. Merged elemental 8-bit images in three color channels were obtained by an additive color model of the normalized levels (values of 0-255) of ^44^Ca (red channel), ^64^Zn (green channel) and ^55^Mn (blue channel).

### Insulin secretion assay

Before the isolation of islets, control and RIP1-Tag2 mice previously anesthetized using 1-2% isoflurane/O_2_ gas mixture (Vetland, Louisville, KY, USA) and intravenously injected with a bolus of MnCl_2_:Bicine solution (75 µmol/kg), analogously to the *in vivo* imaging protocol. Pancreatic islets were isolated by injecting 3-5 mL of a Krebs-Ringer-HEPES (KRH) solution, 120 mM NaCl, 4.7 mM KCl, 1.1mM MgCl_2_, 2.5 CaCl_2_, 10 mM HEPES 0.5 mg/mL Collagenese P, *Clostridium histolyticum*, 1.8 U/ mg lyo (Roche Diagnostics, Indianapolis, IN, USA) and pH 7.4 adjusted with NaOH, via the duodenal duct. After ~6 min of incubation time at 37 °C, the enzymatic digestion was blocked by adding a cold KRH solution, 1% bovine serum albumin (BSA). To represent at the best the heterogeneity of the endocrine pancreas, we tested batches of five islets in triplicates by carefully picking those reflecting at the best the distribution of islets size (Fig. S8C). For the analysis 13-wk old RIP1-Tag2 mice we tested only the heterogeneous islets by avoiding the large tumors (Fig. S9C). Hand-picked islets were kept for 30 min at basal glucose concentration (3 mM) and at room temperature to silence the metabolic activity. Therefore, triplicates of five islets were incubated for 1 h at 37 °C at increasing concentrations of glucose (3, 6, 8, 10, 15 and 30 mM) in KRH solution, 0.5% BSA. The content of insulin in the supernatant was determined by radioimmunoassay using as insulin standards Millipore 8013-K (Merck, Darmstadt, DE). The physiological response of insulin to glucose was assessed by comparing the insulin content measured at stimulated conditions (15 mM) to content measured at basal condition (3 mM).

### Statistical analysis

JMP Software (13.0.0, SAS Institute Inc.) was used for statistical analysis. Differences in the accumulation of the [^64^Cu]Ex4 and Mn between control and transgenic pancreas (Pancreas^Control^ and Pancreas^RIP1-Tag2^) were calculated using a two sample Student's *t*-test and assuming unequal variance between the groups using 0.05 of Alpha level. The comparison of the blood glucose levels between control and RIP1-Tag2 mice was performed by using a nonparametric Rank Sum Wilcoxon's test with 0.05 of Alpha level. The Pearson's coefficients were computed to correlate either the means of co-registered PET and MRI data from the pancreas or voxel-wise by using each individual region using MATLAB's Statistical Tool Box (The MathWorks). Linear regression analysis was performed in the analysis of autoradiography of [^64^Cu]Ex4 and to determine the paramagnetic effect of MnCl_2_:Bicine solution using a 7 T MRI scanner.

## Supplementary Material

Supplementary figures and tables.

## Figures and Tables

**Table 1:** List of the animal groups measured for each *in vivo* and ex vivo experiments.

Groups	PET/ME-MRI		Autoradiography/ MSI		Radioimmuno Assay of Insulin	
	1 h	24 h	1 h	24 h	1 h	24 h
1^13wk^	3/3^a**†**^	-	2/2	-	-	-
2^10-13wk^	-	4/4^b**†**^	-	3/3	-	-
3^10-15wk^	3/7^b*^	6/13^b*^	-	-	-	-
4^8wk^	-	-			3/3	3/3
5^13wk^	-	-	3/2^c^	2/1^c^	3/3	

Animals were measured with ^a^simultaneous or ^b^consecutive PET/MRI setup. Animals that did not receive the initial stimulation with glucose^c^. T_1_ maps were obtained by the acquisition of variable flip angles (4°, 22°^†^; 4°, 14°, 27°^*^).

**Table 2:** Calculated mean ± SD in control and RIP1-Tag2 mice measured at early and late time points.

Overtime quantification PET/ME-MRI signal					
*VOIs*	log_10_(%ID/mL)		log_10_(R_1_)×10^1^		
	1 h	24 h	Pre	1 h	24 h
Pancreas^Control^	0.7 ± 0.1	0.6 ± 0.1	-1.5 ± 0.3	3.0 ± 0.7	0.1 ± 0.6
Pancreas^10-15wk^	1.0 ± 0.3	1.0 ± 0.2	-1.6 ± 0.7	1.2 ± 1.1	-0.1 ± 0.4
Insulinomas^10-15-wk^	1.2 ± 0.3	1.2 ± 0.2	-1.8 ± 0.9	1.2 ± 1.2	0.4 ± 0.5

## Abbreviations

GLP-1R: glucagon-like-peptide-1 receptor
LA-ICP-MSI: laser-ablation induced-by-plasma mass-spectrometry imaging
MRI: magnetic resonance imaging
ME-MRI: manganese enhanced magnetic resonance imaging
PET: positron emission tomography
RIP1: Rat Insulin promotor
STZ: streptozotocin
Tag2: Simian virus 40 large T antigen
T2D: type-2-diabetes
VDCC: voltage-coupled calcium channels
VOI: volume of interest
