# Supplementary material for: PET/MRI enables simultaneous in vivo quantification of β-cell mass and function
Source: Theranostics. 2020 Jan 1;10(1):398–410. doi: 10.7150/thno.33410 (PMC6929626; doi:10.7150/thno.33410)
Supplement: Supplementary file 1 — Supplementary figures and tables. [file thnov10p0398s1.pdf]

Text

Method S1: *in vitro* phantom study

In order to quantify the effect and the concentration of Mn in the pancreas *in vivo*, we first determined the effect of Mn on the T_1 of hydrogens bound to water molecules *in vitro*. An in-house prepared phantom was filled with serial dilutions of a $MnCl_2$:Bicine solution with concentrations ranging from 0.1 to 1 mM (Fig. S5A). Therefore, we calculated the T_1 values of each solution using an inversion-recovery spin echo (IRSE) method as gold standard for the quantification of the T_1. A series of spin-echo pulse sequence were acquired at increasing times of inversion (TI) (50, 150, 300, 600, 1200, 2400, 5500, 9000 ms), TR/TE=10000/12 ms, slice thickness 1.5 mm, one average, matrix size 128×128, BW 25.6 KHz. The regions of interest (ROIs) were drawn for each vial containing the diluted solution of contrast agent and we calculated the T_1 by fitting signal intensity (M_z) using the following equation:

eq. 1 $$M_z = \left| M_0 \left[1 - 2 \left(e^{\frac{-TI}{T_1}} \right) \right] \right|$$

Using a linear regression analysis we assessed the relationship between the increasing concentration of manganese ([Mn]) in the vials of the phantom with the measured T_1: the slope that we obtained from the linear equation (*m*=6.95) represents the index of relaxivity (r_1) of $MnCl_2$:Bicine. This index is proportional to the paramagnetic effect of $MnCl_2$ to shorten the longitudinal relaxation time of hydrogens bound to the water bulk (Fig. S5B) and it was used for the estimation of [Mn] extrapolated from the ΔR_1 maps of pancreas and insulinomas obtained *in vivo*.

In order to perform fast and 3D-T_1 mapping *in vivo*, we optimized *in vitro* a protocol with a relative short acquisition time (<10 min) and high resolution (<1 mm). The variable flip angle

approach (VFA) represented a suitable method allowing the rapid acquisition of large volumes. Therefore, we acquired a set of RF-spoiled GRE-based pulse sequences with multiple pairs of flip angles ranging between 2° to 34°, which were optimized for estimated T_1 values ranging between 500 and 2000 ms. The MR parameters included TR/TE 10/1.5 ms, field of view 30×30×10 mm^3, slice thickness 0.5 mm, matrix size 128×128, BW 86 kHz and by averaging the signal using 2 or 20 number of repetitions (NA). The optimized pairs of flip angles were calculated using an algorithm implemented in the Syngo software in Maplt-Package. The VFA T_1 values of each ROI were obtained by fitting the signal intensity (S_α) acquired using the following equation:

eq. 2 $$S_\alpha = M_0 \frac{(1-E_1)\sin\alpha}{1-E_1\cos\alpha} \quad \text{with} \quad E_1 = e^{-\frac{TR}{T_1}}$$

In the graph are scattered the VFA T_1 values calculated from two flip angles (α=4°, 23°) and with 2 or 20 of number of averages (Fig. 5C-D). The values of T_1 of each ROI are plotted against those values calculated with the IRSE method. By using a combinatorial approach, we tested the accuracy of larger datasets of T_1 values generated by using all the combination (*Ct*) of two or three flip angles (r=2|3). Therefore, we computed the sum squared error and we plotted only the values lower than the 10th quantile (*C*) of each dataset. By evaluating the selected combinations, we observed that T_1 values calculated using three flip angles showed lower squared error compared those with two flip angles. Similar results were also obtained using a higher number of averages (20).

Method S2: Autoradiography of [^{64}Cu]Ex4 for correlation with IHC

Animals were sacrificed and pancreata were extracted, formalin-fixed and paraffin-embedded. Sequential pancreatic sections of 10 µm thickness were cut, mounted on glass slides and dried for 1.5 h at 60 °C. For the 1-h-uptake AR, sections were exposed to a phosphor screen (Fuji Film BAS-SR 2025, Raytest, Straubenhardt, Germany) at room temperature for 66 h. Autoradiography images were scanned with a radioluminography laser imager (Fuji Film BAS 1800 II System, Raytest) and analyzed using Aida Image Analyzer Software (Raytest). 24-h-uptake AR slides were exposed to a 35×43 cm² Storage Phosphor Screen (445SI, Molecular Dynamics, Sunnyvale, CA, USA) at room temperature for 66 hours. Scanning of the phosphor imaging plate was performed using a Storm 840 scanner (STORM 840, Amersham Biosciences, Amersham, UK) with a spatial resolution of 50x50 μm^2 and analyzed using ImageJ software [1] (US NIH, Bethesda, Maryland, USA).

Method S3: Immunohistochemical (IHC) staining of insulin

A series of 4 µm thin sections adjacent to the ones used for autoradiography were stained for insulin content. After deparaffination, antigen retrieval was performed with 10 mM citrate, pH 6.0, for 10 min at 96 °C. Blocking of endogenous peroxidase was performed by incubating in 3% H2O2 in PBS for 10 min at room temperature. Subsequently, sections were blocked by 20% normal goat serum for 30 min at RT, followed by staining with guinea pig anti-insulin (1:3000; A0564; DAKO, Agilent, CA, USA) for 60 min at room temperature. After three washing steps with PBS, the sections were incubated with goat-anti-guinea pig-HRP (1:1000; A18775, Thermo Fisher, MA, USA) for 30 min at room temperature in the dark. Sections were counterstained with hematoxylin, and images were acquired using a VisionTek Live

Digital Microscope (Sakura Finetek, Alphen aan den Rijn, The Netherlands) and VisionTekViewer software v 2.6 (Sakura Finetek).

Supplementary Figures

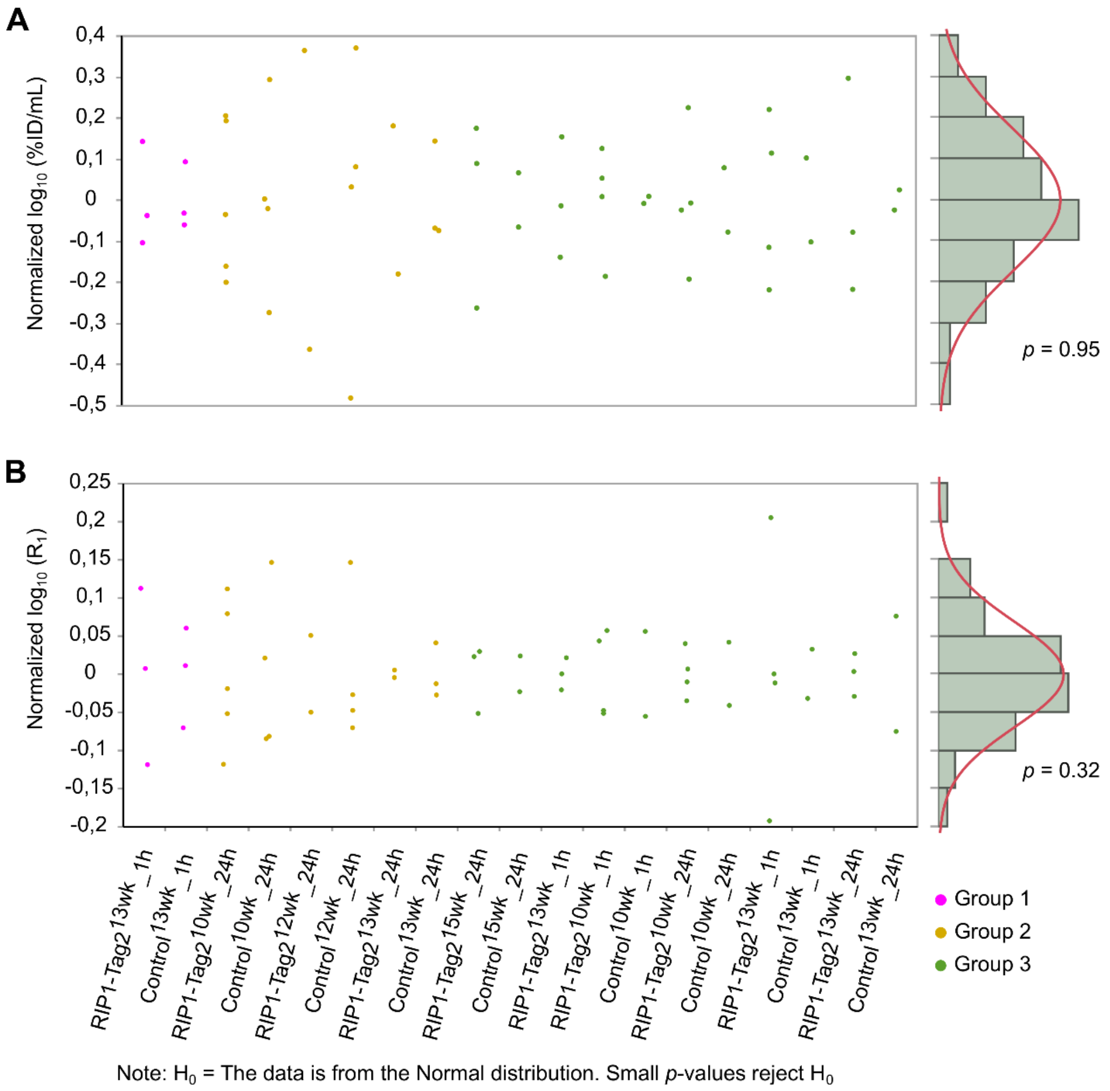

Fig. S1 Analysis of the distribution of PET/MRI data

In the graphs are plotted the **(A)** MRI and **(B)** PET data obtained from each evaluated group of mice. The quantified values were normalized by subtracting the mean values calculated within the group of mice measured at different experimental conditions, which included the strain of animals (Control/RIP1Tag2), the imaging time point (1 h or 24 h) and the age of animals (ranging between 10 to 15 wk). The histograms display the respective distributions of the normalized data, whose normality was tested using a Shapiro-Wilk test.

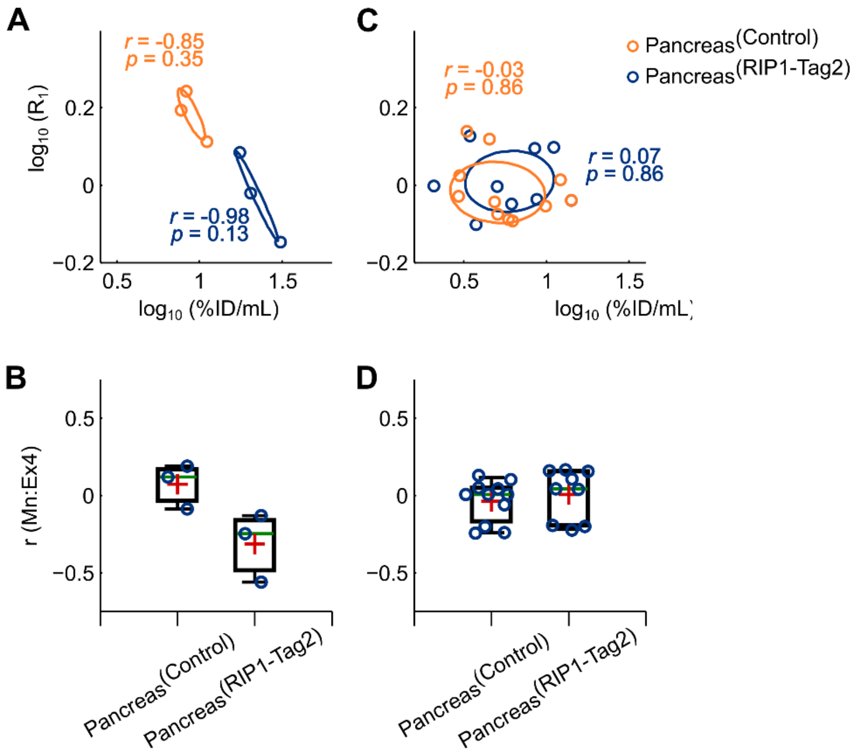

Fig. S2 Group and spatial correlation of PET/ME-MRI signal revealed differences in the accumulation Mn and radiolabeled Ex4 overtime.

The graph depicts the medians of $\log_{10}$(%ID/mL) values plotted against the medians of $\log_{10}(R_1)$ calculated from the VOIs with the respective Pearson's r coefficients and *p* values at **(A)** 1 h and **(B)** 24 h for the control (*r*=0.47, *p*=0.34, dark orange circles) and RIP1-Tag2 mice (*r*=0.14, *p*=0.66, dark blue circles): the ellipses contour the covariance of the two variables. Boxes represent the 1st, 2nd (green line) and 3rd quartile as well as the mean (red crosses) of the *r* coefficients computed voxel-wise from each VOIs acquired at **(C)** 1 h and **(D)** 24 h, respectively.

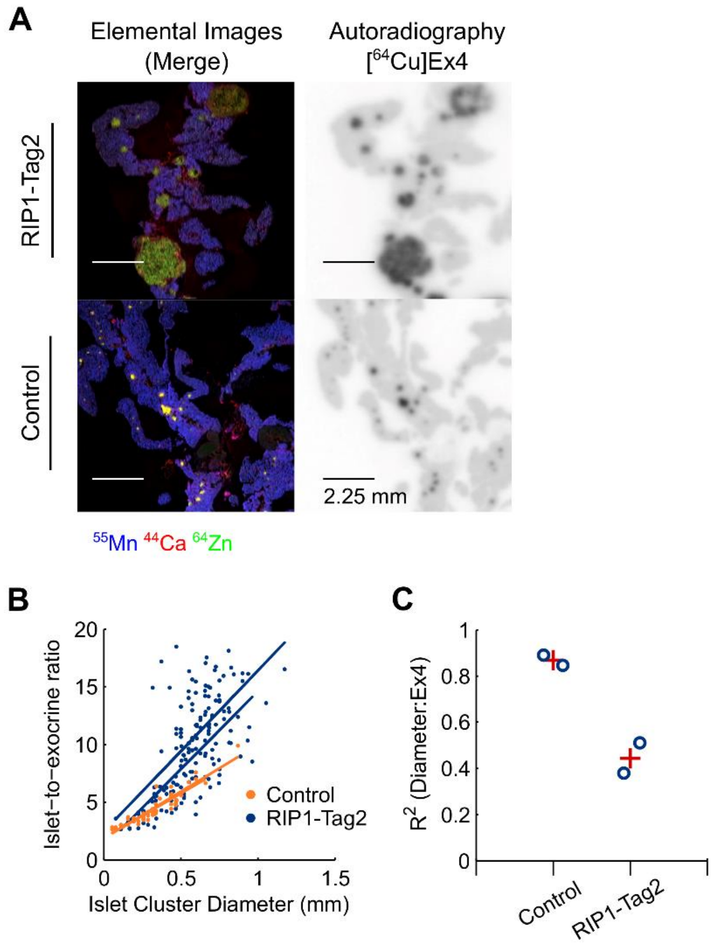

Fig. S3 Merged elemental images and autoradiography of [^{64}Cu]Ex4 revealed high and unspecific binding of ^{55}Mn in the exocrine pancreas measured at early time point (1 h).

(A) Merged images of ^{55}Mn, ^{44}Ca and ^{64}Zn were reconstructed and compared with the autoradiography of [^{64}Cu]Ex4 to determine the co-localization of Mn and the PET tracer 1 h after the co-injection of probes. The cryosections of pancreas were obtained 1 h after injection of radioactive [^{64}Cu]Ex4 solution (~0.12 MBq/g) and scanned at high spatial resolution (50 µm) following analysis biometals by LA-ICP-MS imaging. Merging of elemental images allowed the identification of diffuse spot-like areas (yellow spots) reflecting the co-localization of ^{44}Ca (red channel) and ^{64}Zn levels (green channel). Scale bar are set to 2.25 mm. The distribution of endogenous calcium and zinc was in excellent agreement with the areas positive for the accumulation of [^{64}Cu]Ex4 (dark spots) in the native and tumorigenic pancreas. In contrast, high levels of ^{55}Mn (blue channel) were localized only in the surrounding exocrine pancreas. (**B**-**C**) The analysis of autoradiography indicates a positive relationship between the PET tracer uptake and the islet cluster size calculated for control (dark orange dots) and transgenic pancreas (dark blue dots). The R^2 coefficients calculated by linear regression analysis as well as the mean values (red crosses) are shown for each group.

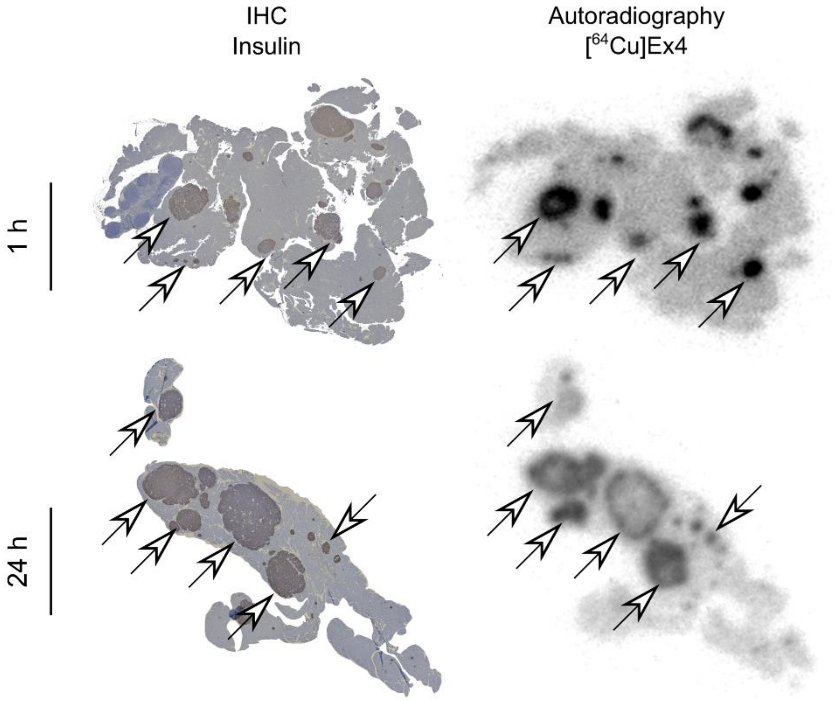

Fig. S4 Insulin staining and autoradiography of the pancreas sections of RIP1-Tag2 mice

The figure shows representative pancreatic sections stained for insulin using immunohistochemistry (IHC) and corresponding autoradiography slices obtained from adjacent pancreatic sections which were isolated 1 h and 24 h after the i.v. administration of the [^{64}Cu]Ex4 tracer solution. The white arrows indicate the presence of pancreatic insulinomas with elevated insulin content and high accumulation of PET tracer.

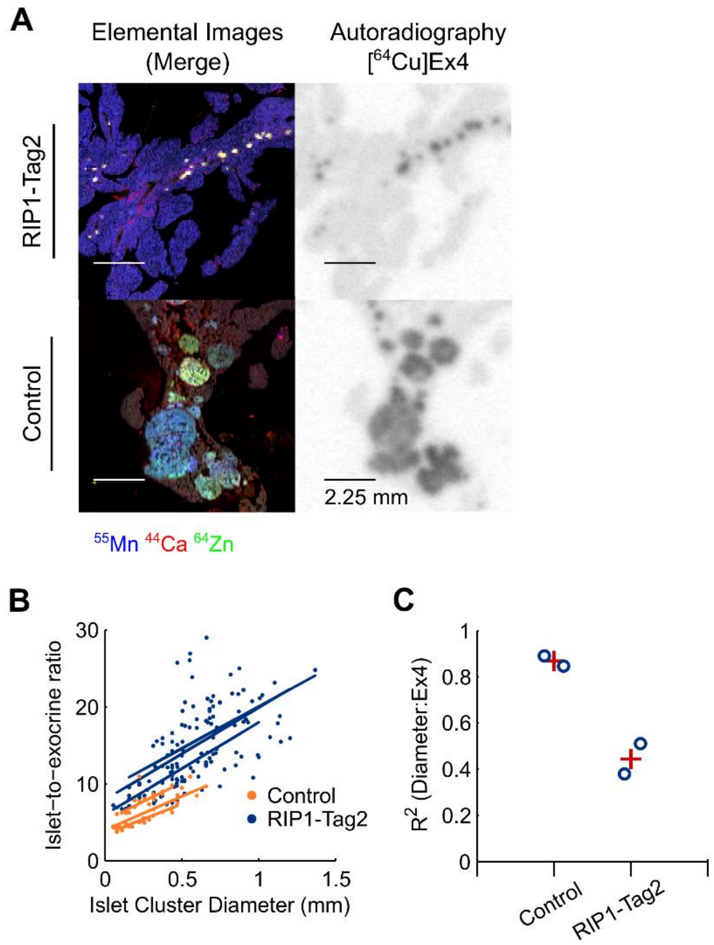

Fig. S5 Merged elemental images revealed high spatial correlation between paramagnetic ^{55}Mn and [^{64}Cu]Ex4 measured at late time points (24 h).

(**A**) Merged elemental images compared with the autoradiography of [^{64}Cu]Ex4 obtained at 24 h after injection. Diffuse spot-like areas identified by elemental imaging (white spots) and autoradiography (dark spots) indicate a direct co-localization of ^{44}Ca (red channel), ^{64}Zn (green channel) and ^{55}Mn (blue channel) in tissue areas with increased accumulation of [^{64}Cu]Ex4 in both groups. Scale bar are set to 2.25 mm. (**B**-**C**) Linear regression analysis of autoradiography indicates a positive relationship between the late retention of PET tracer uptake and size of native islets and insulinomas. R^2 coefficients and the respective mean values (red crosses) are plotted for both groups.

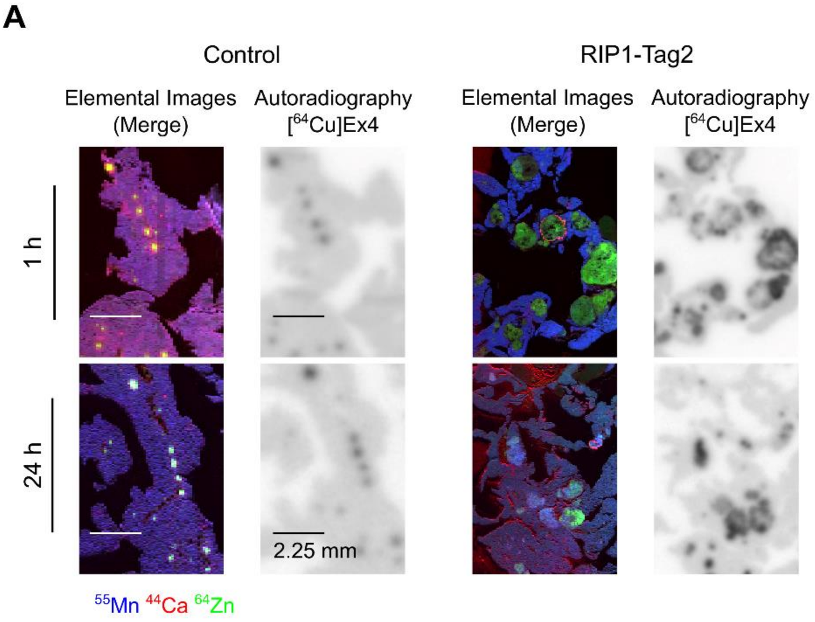

Fig. S6 Glucose stimulation does not influence the transitory binding of Mn by the exocrine pancreas.

Ex vivo of pancreas sections was performed without the pre-injection of glucose. Merged elemental images produced by additive color model of ^{44}Ca (red channel), ^{64}Zn (green channel) and ^{55}Mn (blue channel) are compared with the autoradiography of pancreas sections were performed at an early (1 h) and a late time point (24 h) after the co-injection of Mn-based contrast agent and the PET tracer. Scale bar are set to 2.25 mm. The black arrows indicate the co-localization of ^{64}Zn and ^{44}Ca levels (yellow spots) and the respective accumulation of [^{64}Cu]Ex4 in the same tissue areas 1 h after the injection. Diffuse spots-like areas localized throughout merged elemental images (white spots) and the autoradiography (dark spots) reflect the co-localization of ^{64}Zn, ^{44}Ca and exogenous ^{55}Mn and the accumulation of PET tracer.

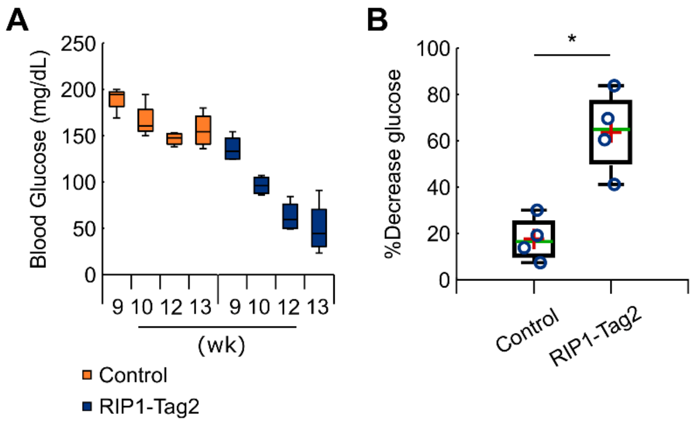

Fig. S7 Normoglycaemia is progressively impaired over the period 10- 13 wk of age of RIP1-Tag2 mice.

(A) Boxplot analysis of the means of blood glucose levels measured during the progression of insulinomas (10 to 13 wk of age); **(B)** Comparison of the percentage decrease in the blood glucose concentration in the period between 9 to 13 of aged-matched control and RIP1-Tag2 mice (Z=2.16, **p*=0.03).

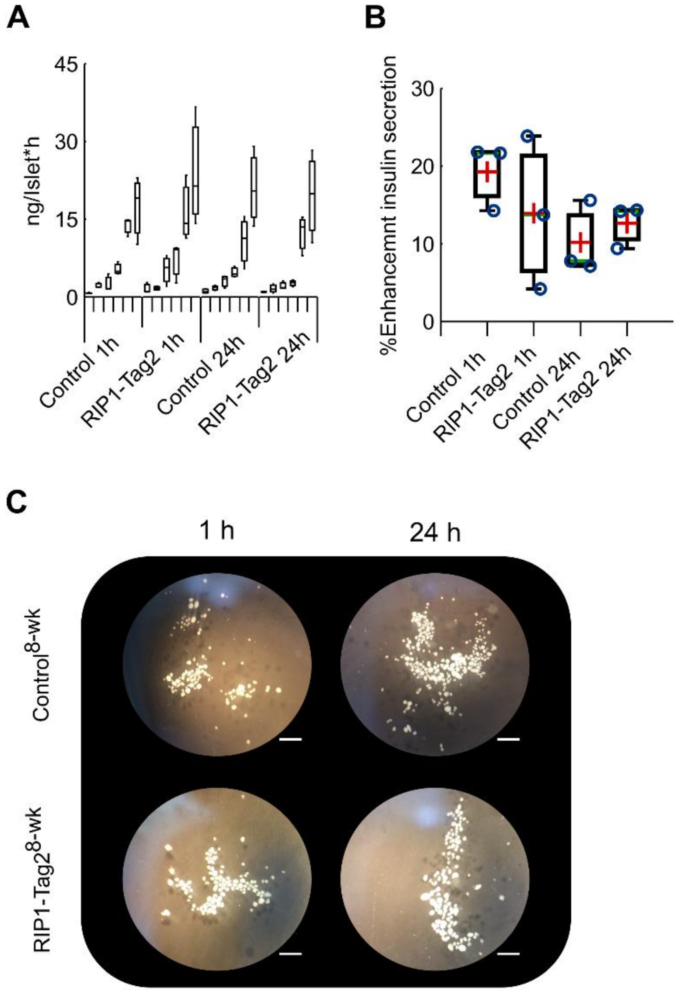

Fig. S8 Insulin secretion response to glucose in 8-wk old control and RIP1-Tag2 mice.

(A) The boxplots depict the means insulin concentration measured at different glucose conditions (3, 6, 8, 10, 15 and 30 mM). **(B)** The enhancement of insulin secretion is calculated between the content of insulin measured at basal (3 mM) and the stimulating glucose conditions (15 mM) of each individual mouse. Boxes represent the 1st, 2nd (green line) and 3rd quartile as well as the mean (red crosses) of each distribution. The figures (**C**) depict the islets isolated from 8-wk-old control and RIP1-Tag2 mice and visualized by optical microscopy with lens magnification 40x; scale bars are settled at 2.25 mm.

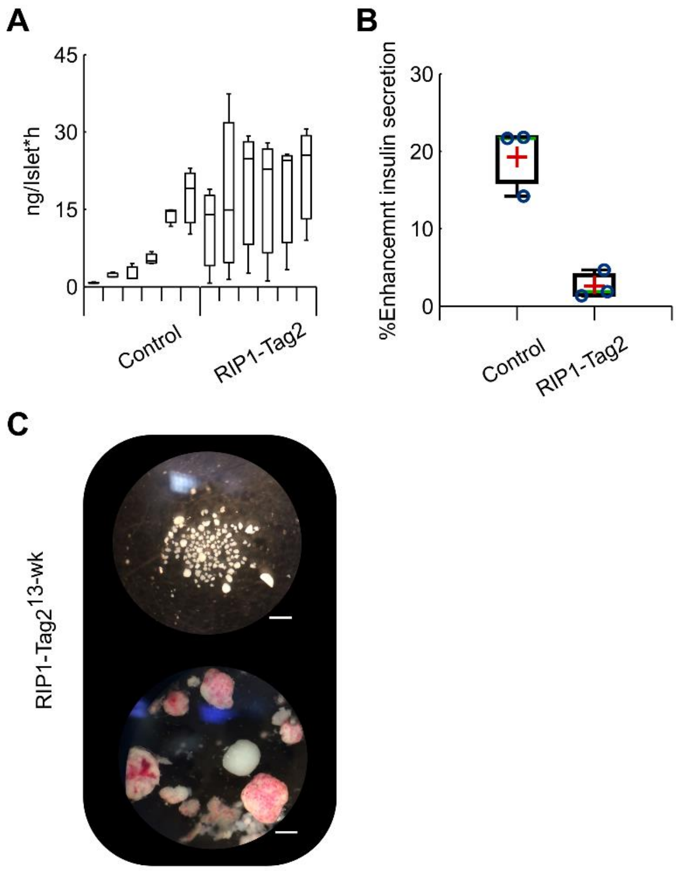

Fig. S9 Insulin secretion response to glucose in 13-wk old control and RIP1-Tag2 mice.

(A) The boxplots show the interquartile range of insulin content measured at different glucose conditions (3, 6, 8, 10, 15 and 30 mM). **(B)** Boxes show the enhancement of insulin secretion: the 1st, 2nd (green line) and 3rd quartile as well as the mean (red crosses) of each distribution. In the figure (**C**) are depicted the isolated islets of 13-wk-old control and RIP1-Tag2 mice. Scale bars are settled at 2.25 mm.

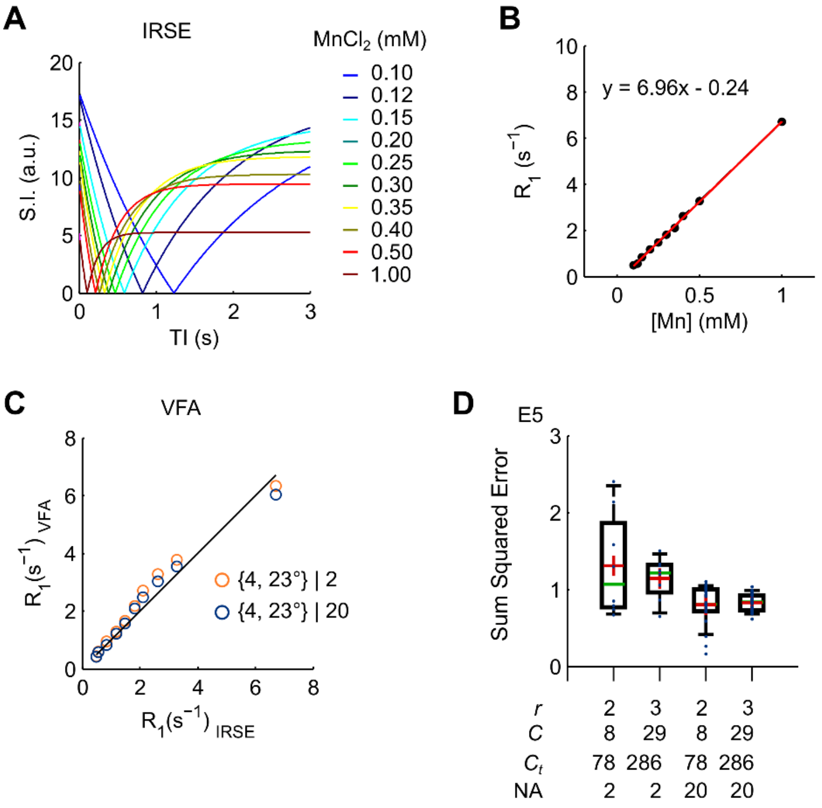

Fig. S10 Three and two angle VFA T_1 mapping method showed high precision to quantify a wide range of T_1 values.

Rapid quantification of volumetric T_1 maps is achieved by using VFA approach using two and three flip angles. **(A)** Fitted signal intensities for each vial filled $MnCl_2$:Bicine solution with concentrations ranging from 0.1 to 1.0 mM were plotted against the time of inversion. **(B)** IRSE R_1 values and increasing MnCl2:Bicine solution of the respective measured vials were plotted and fitted (red-line) by linear regression analysis. **(C)** The graph depicts the R_1 values of ROIs, which were obtained by fitting images acquired using two flip angles (4°, 23°) and 2 or 20 of averages (NA). **(D)** The boxes depict the 1st, 2nd (green line) and 3rd quartile and the means (red crosses) of the sum squared errors (SSE) of each subset (*C*) which were selected from all the possible combinations (C_t) of two and three flip angles (r=2|3), respectively.

Tables

Supplementary Table. 1

Means and Standard Deviations of PET/ME-MRI quantification

	log_{10}(%ID/mL)		$log_{10}(R_1)\times10^1$			[Mn] ($mM\times10^{-1}$)	
VOIs	1 h	24 h	Pre	1 h	24 h	1 h	24 h
$Pancreas^{Control}$	0.8 ± 0.1	0.6 ± 0.1	-1.5 ± 0.3	2.7 ± 0.3	1.0 ± 0.8	1.8 ± 0.2	0.6 ± 0.2
$Pancreas^{10\text{-}wk}$	0.8 ± 0.1	0.8 ± 0.2	-1.6 ± 0.4	1.5 ± 0.6	0.0 ± 0.2	1.2 ± 0.2	0.4 ± 0.1
$Insulinomas^{10\text{-}wk}$	1.0 ± 0.2	1.1 ± 0.1	-2.3 ± 0.3	1.8 ± 0.2	0.5 ± 0.5	1.4 ± 0.2	0.8 ± 0.2
$Pancreas^{13\text{-}wk}$	1.2 ± 0.2	1.1 ± 0.3	-1.6 ± 1.0	0.9 ± 1.6	0.1 ± 0.3	1.0 ± 0.5	0.5 ± 0.4
$Insulinomas^{13\text{-}wk}$	1.3 ± 0.3	1.3 ± 0.1	-1.4 ± 1.1	0.6 ± 1.1	1.6 ± 0.2	0.9 ± 0.4	0.3 ± 0.4

Group means ± standard deviations of the medians log_{10} %ID/mL, R_1 values, and [Mn] calculated from the VOIs of the pancreata of control and RIP1-Tag2 mice as well as the detected insulinomas measured with MRI and PET before (n=3/4), 1 h (n=3/4) and 24 h (n=3/3) after the co-injection of Mn-based MR contrast agent and the PET tracer.

References:

1. Schneider, C.A., Rasband, W.S. & Eliceiri, K.W. NIH Image to ImageJ: 25 years of image analysis. *Nat Methods* **9**, 671-675 (2012).
